# Characteristics and job satisfaction of general practitioners using complementary and alternative medicine in Germany - is there a pattern?

**DOI:** 10.1186/1472-6882-11-131

**Published:** 2011-12-19

**Authors:** Stefanie Joos, Berthold Musselmann, Joachim Szecsenyi, Katja Goetz

**Affiliations:** 1 Department of General Practice and Health Services Research, University Hospital Heidelberg, Voßstrasse 2, 69115 Heidelberg, Germany; 2 Practice of Family Medicine, Academic Teaching Practice, University of Heidelberg, Hauptstrasse 120, 69168 Wiesloch, Germany

## Abstract

**Background:**

The use of Complementary and Alternative medicine (CAM) has increased over the past years. In Germany, many general practitioners (GPs) use CAM in their daily practice. However, little is known about possible differences of GPs using CAM compared to GPs not using CAM. The aim of the study was to explore differences in personal and practice characteristics, work load and job satisfaction of GPs depending on their use of and attitude towards CAM. Furthermore, predictors for CAM use should be explored.

**Methods:**

A questionnaire was developed based on qualitatively derived data. In addition, a validated instrument assessing job satisfaction was included in the questionnaire, which was sent to 3000 randomly selected GPs in Germany.

**Results:**

1027 returned the questionnaire of which 737 indicated to use CAM in daily practice. We found that GPs using CAM are more female, younger and have a trend towards a healthier life style. Their practices have higher proportions of privately insured patients and are slightly better technically equipped with ultrasound. GPs with a positive attitude had significant better values within the job satisfaction scale and lower working hours per week compared to GPs with neutral/negative attitude. Significant predictors for CAM use were a positive attitude towards CAM, holding a special qualification in CAM, own CAM use and the availability of an ultrasound in practice.

**Conclusions:**

The identified differences suggest that those GPs using and believing in CAM have a different medical orientation and approach which in turn may influence their job satisfaction. With this finding CAM use turns out to be a relevant factor regarding job satisfaction and, with this, may be a possible lever to counteract the growing dissatisfaction of GPs in Germany. This finding could also be important for designing strategies to promote the recruitment of young doctors to general practice.

## Background

The increasing popularity of Complementary and Alternative Medicine (CAM) is associated with an ongoing debate of integrating those therapies into mainstream health care. In Germany, at the end of 2009 a number of nearly 63.000 CAM postgraduate CAM qualifications were registered among all 407.000 physicians in Germany, thereof 43.000 related to physicians working in outpatient ambulant care [1]. Many general practitioners (GPs) are providing CAM in their daily practice without having any CAM certification. Specific CAM methods are covered by the statutory health insurance, namely physiotherapy, chiropractic, classic naturopathy, homeopathy (to a very small extent) and, acupuncture in patients with knee and lumbar pain. However, most CAM methods are only reimbursed by private health insurances or have to be paid by patients themselves. The reasons for the increasing numbers of GPs providing CAM may be complex. There could be economic or organizational reasons as well as reasons regarding the attitude or job satisfaction.

Job satisfaction of physicians is an important issue because poor satisfaction is associated with suboptimal healthcare delivery and poor clinical outcomes [2]. In a systematic literature review it has been shown that job satisfaction of GPs decreases with the number of working hours and low income and increases with contact to other colleagues and more variety in job [3]. The latter could be one possible reason that more and more GPs integrate CAM in their every-day practice, in Germany about 60% [4]. By the GPs, the provision of CAM might be seen as a way of "escaping the treadmill" of budgeted reimbursement schemes leading to exploding patient contacts with decreasing consultation times accompanied by high physical and mental burden for the GPs. This development substantially contributes to the situation that many western countries face a shortage of physicians particularly in the field of primary care. A situation which seems much more dramatic in Germany compared to other countries [5].

In view of this, the aim of the presented study was to explore whether there are differences in personal characteristics, practice characteristics, work load and job satisfaction of GPs in dependence of their use of CAM in every-day practice.

## Methods

### Design and participants

The presented study was designed as a cross-sectional survey with a nationwide random sample of GPs (included were 'Fachaerzte für Allgemeinmedizin' and 'praktische Aerzte'). In March 2007, questionnaires were sent to 3000 randomly selected GPs. The recruiting process and the development of the questionnaire are described elsewhere [4,6].

Upon request the ethics committee of the University of Heidelberg informed us that ethics committee approval would not be necessary for this study (email dated 15 August 2011).

### Measures

GPs were asked to answer questions about gender and age, health behaviour, location of practice, working hours per week, average number of patients per day, the proportion of privately insured patients per practice and the average time for consultation and the availability of ECG, ultrasound and spirometer. The health behaviour of GPs was evaluated with two questions, one to work out and one to smoking. Furthermore the body mass index (BMI) was calculated using height and weight. Furthermore, GPs were asked to indicate whether they use CAM in every-day practice (no/yes) and whether they have a positive, negative or neutral attitude towards CAM (5-point-Likert scale with 1 = very negative to 5 = very positive). Finally, job satisfaction was measured according to a modified version of the Warr-Cook-Wall job satisfaction scale developed by Warr et al. (5-point Likert scale with 1 = extreme dissatisfaction to 5 = extreme satisfaction) [7]. Cronbach's α of the modified job satisfaction scale in this study was 0.82.

### Data analysis

Continuous data were summarized using means and standard deviations. Categorical data are presented as frequency counts and percentages, whereas responses of the 5-point Likert scales to the attitude towards CAM were summarized within three categories (positive, neutral, negative). Physician characteristics and practice characteristics are presented as numbers, proportions or means with standard deviation. Group comparisons between CAM users and non-users were performed using the Chi-square test for categorial variables and t-test for metric variables resp. Job satisfaction and work load were compared between CAM users and non-users as well as between GPs indicating a positive, neutral or negative attitude towards CAM with list wise exclusion of missing data. Group comparisons between CAM users and non-users are presented as mean differences and corresponding 95% confidence intervals (CI). Group comparisons regarding the attitude toward CAM were done using ANOVA with Bonferroni correction for post-hoc tests with list wise exclusion of missing data. Predictors for CAM use were calculated by binary logistic regression analysis. Gender, age, years of practice, smoking status, availability of ultrasound, job satisfaction items, attitude towards CAM and own CAM use showed significant correlation and were included as covariates in the regression analysis.

The analyses were performed using SPSS version 18.0 (SPSS Inc., Chicago IL, USA). An alpha level of P ≤ 0.05 was used for tests of statistical significance. However, as this was an exploratory analysis, *p *values are only descriptive in nature.

## Results

Out of 3000 questionnaires handed out, 1027 were returned, which resulted in a response rate of 34.0%. The mean age of respondents was 51 years and 40% were female physicians. 737 of the 1027 GPs completing the questionnaire responded to the question 'Do you use CAM in your every-day practice?' with 'yes', 141 responded 'no' and 149 did not answer the question. 503 GPs indicated a positive, 127 indicated a negative and 280 a neutral overall attitude towards CAM. Detailed descriptive analyses are described elsewhere [4].

### GPs' personal and practice characteristics

The personal characteristics of the participating GPs were compared between CAM users and non-users (Table 1). Table 1 shows significant differences regarding gender, age, years of practice, the existence of additional CAM qualifications and own use of CAM between CAM users and non-users. Regarding life style factors, a significant difference in smoking status can be observed and slightly better values for BMI and sport habits.

**Table 1:** Physician characteristics*

	CAM use n = 737	No CAM use n = 141	p-value
Gender, n (%)			0.02
female	319 (43.3)	46 (32.6)	
male	411 (55.8)	95 (67.4)	

Age (years), mean	50.7	53.2	< 0.01

Years of practice, mean	14.7	16.6	0.02

Work out (> 30 min), n (%)			0.18
regular	348 (47.2)	56 (39.7)	
occasional	227 (30.8)	45 (31.9)	
rarely	155 (21.0)	38 (27.0)	

Smoking, n (%)	104 (14.1)	26 (18.4)	0.02

BMI (kg/m^2^), mean	24.6	24.8	0.12

Own use of CAM	487 (66.1)	16 (11.3)	< 0.01

Special qualification for CAM, n (%)	447 (60.7)	17 (12.1)	< 0.01
qualification Acupuncture	277 (37.6)	2 (1.4)	< 0.01
qualification Naturopathy	193 (26.2)	3 (2.1)	< 0.01
qualification Manual medicine	125 (17.0)	6 (4.3)	< 0.01
qualification Homeopathy	77 (10.4)	1 (0.7)	< 0.01
qualification Physical therapy	44 (6.0)	4 (2.8)	0.32
qualification Environmental Medicine	23 (3.1)	6 (4.3)	0.49

*n varies due to missing data

61% of the CAM using GPs indicated to possess a special qualification for CAM with acupuncture being the most common qualification followed by naturopathy and manual medicine. In addition to the presented results in Table 1 we analysed the attitude towards CAM with regard to gender: significantly more female GPs (63.8%) had a positive attitude than male GPs (49.7%).

Table 2 shows the comparison of CAM users and non-users regarding organization and technical equipment of the practice. Significant differences are observable regarding the availability of an ultrasound which is higher in CAM using GPs. Moreover, CAM using practices have a higher proportion of privately insured patients (11.4% vs. 8.5%). No significant differences were observable for the practice type, location of practice and the absolute numbers of patients per quarter.

**Table 2:** Practice characteristics of GPs using/not using CAM*

	CAM use n = 737	no CAM use n = 141	**p-value**^#^
Practice type, n (%)			0.63
Single	365 (49.5)	78 (55.3)	
Group practice	360 (48.8)	62 (44.0)	

Location of practice, n (%)			0.56
City (> 100.00)	196 (26.6)	41 (29.1)	
Medium size town	215 (29.2)	35 (24.8)	
Rural area (< 15.000)	311 (42.2)	62 (44.0)	

Technical equipment of practice, n (%)			
			
ECG	711 (96.5)	134 (95.0)	0.41
			
Ultrasound	475 (64.5)	77 (54.6)	0.03
			
Spirometer	648 (87.9)	120 (85.1)	0.33

Number of patients/per quarter, mean (SD)	1209.4 (598)	1305.7 (677)	0.32

Privately insured patients per practice (%)	11.4	8.5	0.01

SD: standard deviation; *n varies due to missing data

^#^Statistical significances of differences: P ≤ 0.05

A positive attitude towards CAM, own CAM use, being in possession of an additional CAM qualification and availability of an ultrasound were identified as significant predictors for CAM use (Table 3). The other covariates gender, age, years of practice, smoking status, job satisfaction items which were included in the binary logistic regression analysis were not significant.

**Table 3:** Predictors for CAM use

	OR	95% CI	**p-value**^#^
Own CAM use	5.70	(2.89-11.20)	< 0.01

Qualification in CAM	3.89	(2.06-7.29)	< 0.01

Attitude towards CAM	2.68	(1.99-3.61)	< 0.01

Ultrasound scan	2.26	(1.34-3.82)	0.02

Nagelkerke's R^2 ^= .472

^#^Statistical significances of differences: P ≤ 0.05

### Job satisfaction and work load

In both groups GPs were most satisfied with the 'amount of variety in job' (4.1 and 4.0 respectively) and most dissatisfied with their 'temporal workload' (2.4 and 2.6 respectively) but were not significant between both groups. Overall, no significant differences between GPs using/not-using CAM regarding items of job satisfaction and work load were found.

**Table 4:** Job satisfaction and workload of GPs using/not using CAM

Job satisfaction^1^	CAM use Mean (SD)	no CAM use Mean (SD)	Mean difference (95% CI)
Amount of responsibility	3.67 (1.01)	3.86 (1.02)	0.19 (-0.01; 0.39)

Amount of variety in job	4.06 (0.86)	4.03 (0.90)	0.03 (-0.21; 0.14)

Hours of work	2.40 (1.21)	2.63 (1.19)	0.23 (-0.01; 0.47)

Physical working condition	2.66 (1.10)	2.81 (1.11)	0.15 (-0.07; 0.36)

Income	2.62 (1.09)	2.68 (1.18)	0.07 (-0.16; 0.30)

Recognition for work	3.71 (0.99)	3.69 (1.07)	0.02 (-0.22; 0.19)

Freedom of working method	3.34 (1.19)	3.18 (1.17)	0.16 (-0.40; 0.09)

Colleagues and fellow workers	3.95 (0.81)	3.88 (0.87)	0.06 (-0.23; 0.11)

Overall job satisfaction	3.67 (0.81)	3.64 (0.87)	0.03 (-0.19; 0.14)

**Work load**			

Working hours per week	50.30 (12.47)	48.34 (11.97)	0.96 (-3.33; 1.46)

Number of patients per day, n (%)	49.38 (23.26)	51.21 (21.50)	1.83 (-2.46; 6.11)

Consultation time (minutes)	11.63 (8.52)	10.66 (9.63)	1.21 (-2.84; 0.89)

^1^each item ranges between 1 = extreme dissatisfaction and 5 = extreme satisfaction

SD = standard deviation

Of the 818 GPs providing CAM, 448 indicated to have a positive attitude towards CAM whereas 253 indicated to have a neutral and 117 have a negative attitude.

Based on the hypothesis that there is a difference between CAM users regarding their attitude we assessed job satisfaction and workload of these subgroups. Remarkably, we found several relevant and statistically significant differences (Table 5). With the exception of physical working condition and income GPs with a positive attitude had significant better values within the job satisfaction scale than GPs with neutral or negative attitude. Furthermore, they had significant lower working hours per week and more time per patient.

**Table 5:** Job satisfaction and work load of CAM using GPs comparing attitudes

Items of job satisfaction^1^	Positive CAM attitude (n = 448) Mean (SD)	Neutral CAM attitude (n = 253) Mean (SD)	Negative CAM attitude (n = 117) Mean (SD)
Amount of responsibility^a^	3.72 (1.01)	3.58 (1.04)	3.93 (0.97)

Amount of variety in job^a,b^	4.12 (0.84)	3.99 (0.94)	4.22 (0.81)

Hours of work^a^	2.48 (1.23)	2.31 (1.11)	2.70 (1.23)

Physical working condition^a^	2.66 (1.11)	2.58 (1.05)	2.91 (1.11)

Income	2.62 (1.09)	2.60 (1.05)	2.83 (1.20)

Recognition for work^b^	3.77 (1.01)	3.56 (1.02)	3.75 (1.07)

Freedom of working method^b^	3.42 (1.18)	3.12 (1.12)	3.35 (1.29)

Colleagues and fellow workers	3.98 (0.85)	3.84 (0.84)	3.99 (0.82)

Overall job satisfaction^b^	3.76 (0.79)	3.55 (0.80)	3.68 (0.91)

**Work load**			

Working hours/week (hrs)	49.09 (13.57)	50.75 (12.09)	51.64 (11.03)

Number of patients/day^b^	47.97 (23.88)	52.23 (21.19)	52.67 (22.00)

Consultation time (minutes)^b^	12.86 (10.36)	10.01 (5.42)	10.65 (8.93)

SD, standard deviation; ^1^each item ranges from 1 "extreme dissatisfaction" to 5 "extreme satisfaction"

^a^Statistical significances: p ≤ 0.05 for neutral vs. negative

^b^Statistical significances: p ≤ 0.05 for positive vs. neutral

^c^Statistical significances: p ≤ 0.05 for positive vs. negative

## Discussion

German GPs who practice CAM seem to have much in common with GPs not using CAM. However, a few significant differences have been found, in particular regarding personal characteristics. In comparison, CAM using GPs are more female, younger and have a trend towards a healthier life style. Their practices have higher proportions of privately insured patients and are slightly better technically equipped with ultrasound. GPs with a positive attitude had significant better values within the job satisfaction scale and lower working hours per week than GPs with neutral/negative attitude. Significant predictors for CAM use were a positive attitude towards CAM, holding a special qualification in CAM, own CAM use and the availability of an ultrasound in practice.

In further analyses we identified several significant differences between CAM using GPs having a positive attitude and those using CAM in spite of a neutral or negative attitude. Consequently, CAM use itself seems not to be a predictor for a high job satisfaction, but the combination of CAM use and having a positive CAM attitude does.

Studies comparing GPs in dependence of their CAM use are available from Canada, Australia and Switzerland [8-11]. In an Australian survey, CAM practising GPs tended to be male and full time working, but showed no further differences regarding practice form, practice location or patients seen per week [8]. In a Canadian study CAM using GPs were significantly younger, male and working in solo practices. Both studies were smaller ones (< 500 respondents) and published before 2002.

The 2006 published Swiss study from Widmer et al surveyed 650 primary care physicians (response rate 29%) with the following findings: physicians using CAM were more female, working in group practice, had lower consultation rates and a slightly lower workload [9].

These findings are in good agreement with our results. However, only when comparing our findings concerning the workload of CAM users with a positive attitude. A remarkable difference of Switzerland and Germany can be seen in the numbers of homeopathy qualifications being the most common ones among Swiss physicians but only on the fourth position in Germany followed by acupuncture, naturopathy and manual medicine. Another difference is that ultrasound is present to a lower extent in CAM practices in Switzerland. In contrast, in Germany, ultrasound was more present in GP practices using CAM. About the reasons can only be speculated.

The reasons why GPs become involved in CAM are diverse. Often the reasons are very personal and closely linked to certain life experiences [6]. Our data show, that, on the one side, there seems to be a congruency between the own life style (e.g. attitude towards and own use of CAM, non-smoking) and orientation of medical care towards CAM. On the other side, CAM using GPs have significantly more privately insured patients. This could be either a consequence of the fact that the majority of CAM interventions are not reimbursed by statutory health insurances. However, this economic advantage could also be a reason for providing CAM.

In a very simplistic view one could hypothesize that there are two groups of GPs using CAM: the economists and the believers. This hypothesis is supported by our finding that differences regarding job satisfaction and work load only arise when considering GPs attitude in the analysis. It seems that GPs with a congruent attitude and use of CAM are more satisfied with their job. Possibly they may have found their way of "escaping the treadmill" and may have developed a higher sense of coherence [12].

A main strength of our study is the nationwide random sample, but because of the low response rate of 34% our results should be regarded with caution. However, also in the Swiss CAM study they had a comparable response rate with 36% [9] and from preceding studies it is known that the motivation of German doctors to participate in surveys of this type is generally low with rates between 15% and 30% [5]. Comparisons with available data aiming at validating work parameters and demographic parameters at least confirm that respondents were representative of the background population in basic respects such as gender, age and location of practice [1]. Moreover, it seems that GPs using CAM were more cooperative to participate in the survey than GPs not using CAM. This is a common bias in survey studies and should be considered interpreting the results. In addition, this is an exploratory study; significant changes are only descriptive in nature and do not confirm a causative relationship

Characteristics of health care providers such as job satisfaction are gaining increasing importance in health services research. On the one hand, job dissatisfaction is a major cause of GPs turnover [13] which can lead to a shortage of GPs. To date, in Germany, there is a continuous decline of the number of GPs, especially of those practicing in the rural parts of the country. On the other hand, physicians' dissatisfaction has not only consequences for physicians but also for patients. There is robust evidence that doctors' feelings of discontent have a significant influence on the quality of patient care [14,15].

At the level of the doctor-patient-relationship, 'time', 'confidence', 'matching', 'balance of power' and 'rituals' seem to play a major role. Believing in their therapies may create a sense of coherence in doctors and may enable them to activate „placebo" in patients and, doing so, to improve therapeutic outcome [16]. Therefore, sufficient time and a coherent medical attitude may be relevant factors for quality of care.

Furthermore, structural characteristics of the practice are important in the assessment of quality in general practice. We have found no significant differences in basic structural characteristics such as practice type, location of practice and number of patients. However, we found that practices of CAM using GPs are slightly better equipped with ultrasound and have more privately insured patients. Whereas the higher proportion of privately insured patients in CAM practices was expected, the association of CAM use with ultrasound is unclear. Since the availability of ultrasound in practice emerged as significant predictor for CAM use this needs to be investigated in further studies.

Another relevant issue in terms of quality of care is the education of the GPs. Only 61% of the CAM using GPs in our sample had an additional CAM qualification. The remaining GPs provide CAM without certified qualification. This is an important point in terms of quality of care and should be a matter of future discussions about the integration of CAM in primary care. In Germany, the present regulation concerning postgraduate education consists of single CAM qualifications for acupuncture, homeopathy etc. Theses regulations should be reconsidered and adapted according to the requirements of the GPs [17]. A possibility would be to establish a postgraduate education scheme combining the most important (evidence-based) CAM methods.

Our findings permit to speculate on the further development of CAM care in the ambulatory setting. With increasing numbers of female doctors and the inclusion of CAM in undergraduate education in Germany [4], it can be supposed that the use of CAM will further increase in the future. Therefore, within this increasing CAM-'market' with a large range in quality of the different methods studies on quality and cost-effectiveness are urgently needed

## Conclusions

Our findings show that GPs using CAM are part of the mainstream medical community. However, the identified differences regarding characteristics of the GPs suggest that those GPs using and believing in CAM have a different medical orientation and approach which in turn may influence their performance. This may be a relevant finding also considering possible strategies to recruit more young doctors for the field of primary care.

## Competing interests

The authors declare that they have no competing interests.

## Authors' contributions

SJ and BM carried out and participated in the design of the study. SJ and KG performed the statistical analysis and drafted the manuscript. BM and JS made contributions to the manuscript. All authors read and approved the final manuscript.

## Pre-publication history

The pre-publication history for this paper can be accessed here:

http://www.biomedcentral.com/1472-6882/11/131/prepub
